# Special Issue “Working Group in Sports Medicine”

**DOI:** 10.3390/jfmk10010012

**Published:** 2024-12-31

**Authors:** Giuseppe Musumeci

**Affiliations:** 1 Department of Biomedical and Biotechnological Sciences, Section of Anatomy, Histology and Movement Science, School of Medicine, University of Catania, 95123 Catania, Italy; giuseppe.musumeci@unict.it; 2 Research Center on Motor Activities (CRAM), University of Catania, 95123 Catania, Italy

## 1. Introduction

The Special Issue “Working Group in Sports Medicine” has been successfully concluded, offering significant contributions to the growing discourse on sports medicine as a dynamic and interdisciplinary field. This collection brings together a range of innovative research findings and methodologies aimed at enhancing physical fitness, preventing injuries, and improving the management and rehabilitation of sports-related conditions. Sports medicine is a branch of medicine concerned with physical fitness and the treatment and prevention of sports- and exercise-related injuries [1]. Its evolution in healthcare occurred around the end of the 20th century and underscores its critical importance in contemporary sports science [2]. Over the years, the discipline has expanded to encompass a variety of specialized fields, including anatomy, biomechanics, psychology, physiology, and kinanthropometry, as well as advanced training approaches to talent identification and performance analysis [3]. This Special Issue addresses some of the most pressing challenges in sports medicine and sports science, providing insights into the prevention and rehabilitation of injuries associated with exercise and physical activity. By incorporating interdisciplinary perspectives, this Special Issue highlights advancements in posturometry, biochemistry, and sports psychology, alongside novel strategies for injury management and health optimization. This Special Issue aims to bridge some of the gaps between theoretical knowledge and practical application in the field of sports medicine. A total of 16 manuscripts were accepted, encompassing diverse topics and offering valuable contributions to both academics and practitioners in the pursuit of safer and more effective practices in sports and exercise.

## 2. Overview of Published Articles

The contributions herein range across experimental research, systematic reviews, and methodological advances, offering both theoretical insights and practical applications to improve health and performance outcomes. A key theme that unites these studies is an emphasis on individualized, targeted interventions and the exploration of interdisciplinary approaches to address complex challenges in the field. One of the highlights is the introduction of a Modified Isoinertial-Based Ruffier Test, which demonstrated equivalence to the classic test in estimating VO2max, but with the added benefit of being shorter and more practical [4]. This development could make fitness assessments more accessible and efficient for diverse populations. Similarly, in the realm of orthopedic care, research into blood-transfusion risk factors in elderly patients undergoing intramedullary nailing for femoral neck fractures identified preoperative hemoglobin levels and surgery duration as critical predictors [5]. These findings underscore the importance of pre-surgical optimization and risk stratification to improve outcomes in vulnerable populations. Furthermore, in the field of anterior cruciate ligament injuries, an MRI-based evaluation of three autografts used in ACL reconstruction revealed that the quadriceps tendon has a larger cross-sectional area and perimeter than the other options [6]. These findings offer valuable guidance to clinicians in selecting the most appropriate grafts for specific patient needs, potentially improving surgical outcomes and recovery trajectories. Rehabilitation strategies also take center stage in this issue. A combined intradialytic exercise program improved functional capacity and body composition in kidney transplant candidates, demonstrating the transformative potential of targeted aerobic and resistance training for patients undergoing hemodialysis [7]. Similarly, constraint-induced movement therapy has been shown to improve motor exploration and variability in spinal cord injury patients, underscoring the critical role of specific challenges in promoting neural and functional recovery [8]. In contrast, a study on the application of Kinesio taping in healthy soccer players revealed no immediate performance benefits, raising questions about its efficacy in enhancing athletic function [9]. However, a different therapeutic approach—mirror therapy—proved beneficial for ACL-injured female football players, significantly improving psychological readiness and reducing pain [10]. Surgical progress was also examined, with intraoperative load bearing during total knee arthroplasty shown to improve walking parameters, although these improvements did not translate into differences in clinical outcomes [11]. This result underscores the nuanced relationship between functional measures and patient-perceived benefits in orthopedic interventions. In addition, athletes with previous hamstring injuries showed altered muscle architecture, particularly shorter fascicle length in the hamstring, indicating potential markers for injury prevention and individualized rehabilitation strategies [12]. The long-term health impacts of sports participation have also been explored. Research linking repeated head trauma in retired athletes to slower motor response times suggests that motor processing speed may be a more sensitive indicator of cumulative neurological damage than accuracy alone [13]. Meanwhile, the autopsy analyses of premature deaths among bodybuilders have revealed abnormal cardiac hypertrophy potentially associated with anabolic steroid use, underscoring the need for greater awareness of the cardiovascular risks related to the use of these substances in this population [14]. Public health considerations emerged in a study of vaccination practices among low-level soccer players in Greece, which revealed inadequate immunization strategies due to unclear guidelines [15]. This finding calls for better education and structured protocols to protect athletes from preventable diseases. It is also interesting to note that a comparison between opera singers and athletes revealed the “conditioning” effects of prolonged vocal training, with both groups showing comparable cardiac performance metrics [16]. This unexpected result expands our understanding of how different forms of physical and physiological training can impact the heart. Finally, systematic reviews have highlighted effective rehabilitation strategies for specific conditions. Interventions targeting the scapulothoracic complex were effective in the management of subacromial impingement syndrome and frozen shoulder [17], while resistance training demonstrated improvements in strength, balance, and mobility in lower extremity amputees [18]. These results underscore the value of targeted, condition-specific approaches to optimize rehabilitation outcomes. These studies illustrate the multifaceted nature of sports medicine, mixing physiological and psychological insights with innovative methodologies. They reinforce the importance of individualized care, interdisciplinary collaboration, and ongoing exploration to meet the diverse needs of athletes and patients.

## 3. Conclusions

The studies presented in this Special Issue highlight the multifaceted nature of sports medicine and its critical role in improving health outcomes, optimizing performance, and guiding rehabilitation strategies. Based on the research included, several promising directions for future studies emerge, paving the way for advancements in the field. Future research should further improve these methods, focusing on biomechanical, physiological, and psychological variability to maximize outcomes and minimize injury risks. Interdisciplinary collaboration, also based on new techniques and strategies, continues to be a key avenue for progress. This Special Issue reflects the collective efforts of the authors, reviewers, and editors to explore the dynamic field of sports medicine and science.
